# Remote network for cognitive symptoms derived from tau accumulation in progressive supranuclear palsy

**DOI:** 10.1126/sciadv.aed0348

**Published:** 2026-07-10

**Authors:** Yuki Hori, Hironobu Endo, Kenji Tagai, Yuko Kataoka, Ryoji Goto, Shin Kurose, Yuki Momota, Naomi Kokubo, Chie Seki, Sho Moriguchi, Hitoshi Shimada, Hitoshi Shinotoh, Takahiko Tokuda, Keisuke Takahata, Takafumi Minamimoto, Makoto Higuchi, Toshiyuki Hirabayashi

**Affiliations:** ^1^ Advanced Neuroimaging Center, National Institutes for Quantum Science and Technology, 4-9-1 Anagawa, Inage-ku, Chiba 263-8555, Japan.; ^2^ Department of Psychiatry, The Jikei University School of Medicine, Tokyo 105-8461, Japan.; ^3^ Department of Psychiatry, Keio University School of Medicine, Tokyo 160-0016, Japan.; ^4^ Center for Integrated Human Brain Science, Department of Functional Neurology & Neurosurgery, Niigata University, 1-757 Asahimachidori, Chuo-ku, Niigata 951-8585 Japan.; ^5^ Department of Neuroetiology and Diagnostic Science, Osaka Metropolitan University Graduate School of Medicine, Osaka 545-8585, Japan.

## Abstract

Progressive supranuclear palsy (PSP) is a neurodegenerative disorder with motor and cognitive impairments. Whereas motor symptoms are associated with subcortical tau deposits, the mechanisms underlying the cognitive symptoms remain unclear due to heterogeneous and primarily subcortical distribution of pathological tau deposits. Here, we combined tau-PET (tau positron emission tomography) using a high-contrast probe we developed with a normative connectome in 37 patients with PSP and 48 healthy controls. We found that tau deposition sites functionally connected to a common cortical network that could not be derived from atrophy. This network predominantly overlapped with canonical action-mode and frontoparietal networks, which support adaptive, goal-directed behavior. Whereas the extent of primary tau deposition predicted motor symptoms, the normative connectivity strength from tau deposition sites to the identified cortical network explained the severity of cognitive deficits. These findings suggest a previously unknown mechanism that cognitive, but not motor, deficits in PSP arise from remote effects of tau deposition—independent of atrophy—via convergent connectivity to a common cortical network.

## Teaser

Executive dysfunction in PSP arises from remote effects of tau deposition that disrupt action-mode and frontoparietal networks.

## INTRODUCTION

Progressive supranuclear palsy (PSP) is a neurodegenerative disorder primarily characterized by movement impairments such as oculomotor deficits, postural instability, and akinesia (*1*–*3*). In addition to these motor-related symptoms, PSP is frequently associated with cognitive deficits, especially those related to frontal cortical functions (*4*, *5*). Clinically, PSP is a heterogeneous disorder comprising multiple phenotypic variants beyond the classical Richardson’s syndrome, including PSP with parkinsonism (PSP-P), progressive gait freezing (PSP-PGF), frontal presentation (PSP-F), postural instability (PSP-PI), ocular motor dysfunction (PSP-OM), speech/language disorder (PSP-SL), corticobasal syndrome (PSP-CBS), and primary lateral sclerosis (PSP-PLS) (*3*). Pathologically, PSP is known as a four-repeat tauopathy, where the aggregation of hyperphosphorylated tau proteins is implicated as a major cause of clinical symptoms, accompanied by neuroinflammation and atrophy (*6*, *7*). Tau lesions, neuronal loss, and gliosis have been found primarily in subcortical regions including the subthalamic nucleus, globus pallidus (GP), and substantia nigra (*8*, *9*), and damage to these structures has been associated with motor dysfunction and general disease severity (*2*, *3*, *10*, *11*). Subcortical atrophy—especially in the midbrain tegmentum and the dentate nucleus of the cerebellum—is another notable pathological hallmark of PSP and can be used to distinguish patients with PSP from those with Parkinson’s disease or age-matched healthy controls (HCs) (*12*, *13*). However, the mechanisms underlying the frontal cortex–related cognitive decline in PSP remain elusive as tau deposition or atrophy within the frontal cortex itself is only rarely observed beyond the primary and supplementary motor areas (*13*–*16*).

Neurodegenerative diseases, including PSP, are increasingly being conceptualized as network-based disorders (*17*). The aggregation of pathological proteins in specific brain regions disrupts synaptic function, with damage propagating across interconnected networks (*18*, *19*) and accelerating clinical symptoms (*20*). A recent study showed that normative functional connectivity (FC) from patient-specific tau epicenters—the subcortical region with the highest tau positron emission tomography (tau-PET) signal—predicts the whole-brain pattern of tau deposition in PSP, supporting a model of connectivity-constrained tau propagation (*21*). Meanwhile, reduced FC between the atrophied midbrain and distal regions, including the frontal cortex, has been reported in PSP (*22*). In addition, hypoperfusion (*23*, *24*) and hypometabolism (*25*, *26*) in the frontal cortex have also been observed even in the absence of the corresponding local pathological changes, suggesting that the cognitive dysfunctions in PSP might result from the remote effects of subcortical pathology.

Using normative connectome data, lesion network mapping (LNM) is a powerful approach for identifying brain regions commonly connected from heterogeneous lesion sites that are associated with shared symptoms across patients with stroke. This approach is based on the principle that, in addition to direct local impairments, a lesion in a given brain region also indirectly impairs functions of remote regions connected from the lesion site. LNM has uncovered hidden core regions associated with diverse symptoms across neurological conditions, including visual hallucinations (*27*), delusional misidentifications (*28*), amnesia (*29*), loss of consciousness (*30*), criminal behavior (*31*), and abnormal movements such as freezing of gait and hemichorea-hemiballismus (*32*). Core regions identified through LNM have been shown to align with previously identified neuromodulatory targets for symptom relief (*33*, *34*), further validating its utility. Recently, LNM has been successfully extended to tau deposition–based or atrophy-based analysis in Alzheimer’s disease (AD) (*35*–*37*), demonstrating its potential for identifying common brain locations functionally connected from regions of pathology in spite of across-patient variability in the spatial pattern of pathology in a given neurodegenerative disease. However, tau/atrophy network mapping has not been applied to non-AD tauopathies, and critical comparisons between the influences of local tau deposits/atrophy and their remote network effects on a specific symptom and between the effects of tau deposits and atrophy on the network mapping have not been conducted yet for any neurodegenerative diseases.

**Fig. 1.**
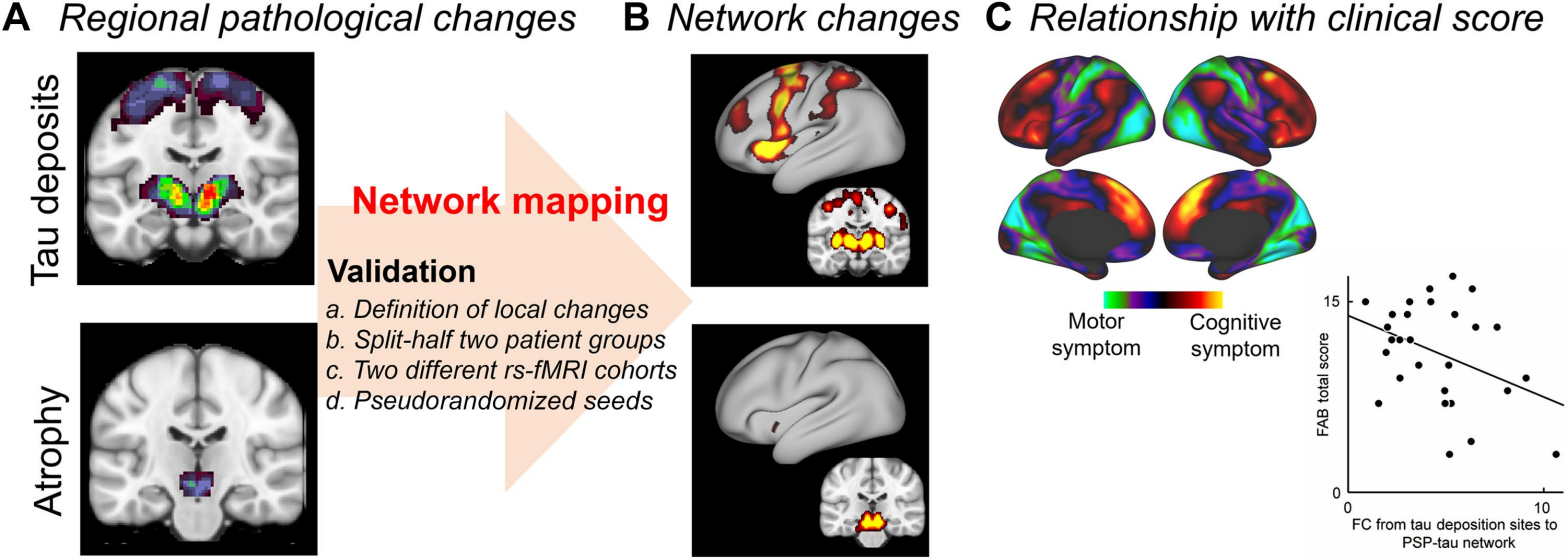
Study overview. Regional and network impairments responsible for the cognitive dysfunctions were examined in patients with PSP. (**A**) Regional impairments caused by tau deposits and atrophy were identified via ^18^F-florzolotau PET and MRI, respectively. (**B**) Common network impairments across the patients were identified using an rs-fMRI database of healthy participants with tau deposition/atrophy sites in individual patients as seed regions. (**C**) Last, we examined whether clinical symptoms of individual patients could be best explained by the degree of local pathologies or their network effects.

In the current study, we hypothesized that a common network extended from tau deposition sites is responsible for the cognitive dysfunctions observed frequently in PSP. Specifically, we first examined whether such common network can be identified for PSP and further investigated whether motor and cognitive impairments in PSP could be attributed to local tau burden or its remote effects exerted on the common extended network. To achieve this, we used ^18^F-florzolotau (also known as PM-PBB3 or APN-1607), a novel PET probe that we recently developed for detecting tau deposits (*38*). This probe provides high metabolic stability and remarkable contrast at the single-patient level not only for AD but also for non-AD tauopathies including PSP, which has been difficult with previous probes (*39*). Postmortem autoradiography and histopathology using this probe have demonstrated robust labeling of both 3R and 4R fibrillar tau lesions, and in vivo PET retention has been shown to correspond to AT8/GB-positive tau pathology in autopsy-confirmed PSP (*38*). In practice, although florzolotau alone cannot distinguish 4R tauopathy from mixed 3R/4R cases, combining it with amyloid PET using Pittsburgh compound B (PiB) enables differential classification. Off-target retention in the choroid plexus has been reported, whereas autoradiographic blocking studies have indicated minimal MAO-A/B cross-reactivity (*38*). By applying network mapping to PET-detected tau deposits in individual patients, we identified common brain regions that are remote but functionally connected from heterogeneous locations of tau deposit and examined their relationships with clinical symptoms (Fig. 1). Our results indicate that a network mechanism underlies the cognitive impairments in PSP and highlight the distinct contribution of remote effects driven by tau burden.

## RESULTS

### Localization of tau deposits and brain atrophy in individual patients with PSP

We analyzed the data from 37 patients (22 males and 15 females) with PSP–Richardson’s syndrome (PSP-RS), which presents the most typical clinical features of PSP (*40*). The mean patient age and disease duration were 70 ± 7.3 years and 3.4 ± 2.4 years (mean ± SD), respectively. Disease severity as assessed by the PSP rating scale (PSPRS) was 40.9 ± 17.4. All patients exhibited tau deposits visualized and quantified with florzolotau PET, without any accumulation of amyloid-β, as determined by PiB-PET (table S1).

**Fig. 2.**
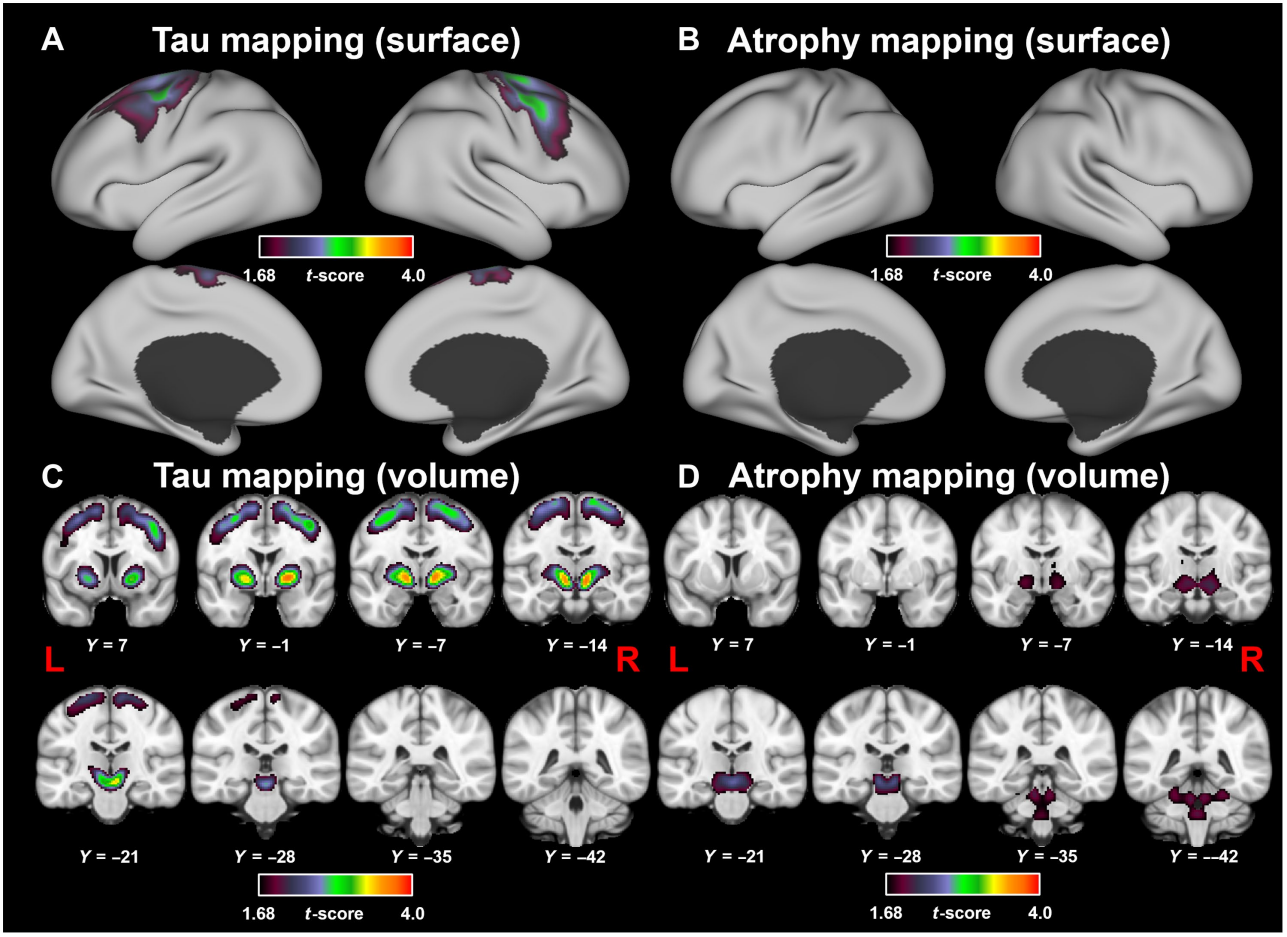
Population maps of tau deposition and atrophy among the patients with PSP. (**A** to **D**) *T*-maps of tau deposition [(A) and (C)] and atrophy [(B) and (D)] among the patients with PSP were presented on the surface (A and B) and volume space [(C) and (D)], respectively. *T*-scores were derived from the unpaired *t* test against HCs and were displayed at a voxel-wise threshold of *P* < 0.05 (uncorrected). Tau deposits were primarily observed subcortically in the GP and midbrain. Moderate accumulation of tau was also detected in motor-related cortical areas in the precentral gyrus. Atrophy was observed in the midbrain and cerebellum.

To identify the locations of tau deposition and atrophy, we first calculated *t*-score maps for florzolotau PET and magnetic resonance imaging (MRI)–based voxel-based morphometry (VBM) images for each patient by comparing their data with those of gender-matched HCs (*n* = 48) (Fig. 2). Across the patients, tau deposits were most frequently observed subcortically in the GP (*P* < 0.0003, unpaired *t* test) and midbrain (*P* < 0.0006), with 62 and 57% overlaps across patients when thresholded at *P* = 0.001, respectively (fig. S1C). In addition, moderate tau accumulation was also detected in motor-related cortical areas in the precentral gyrus (*P* < 0.003, 25% overlap). Meanwhile, brain atrophy was primarily localized to the midbrain (*P* < 0.01, 27% overlap), cerebellum (*P* < 0.03, 27%), and GP (*P* < 0.05, 14%) (fig. S1, B and D). Note that overlap of both tau deposition and atrophy was extremely limited in the frontal cortex, except for some regions in the primary/supplementary motor and premotor areas. Regions with tau accumulation and atrophy for each patient are shown in fig. S2.

### Common extended network derived from tau deposits or atrophy across patients with PSP

**Fig. 3.**
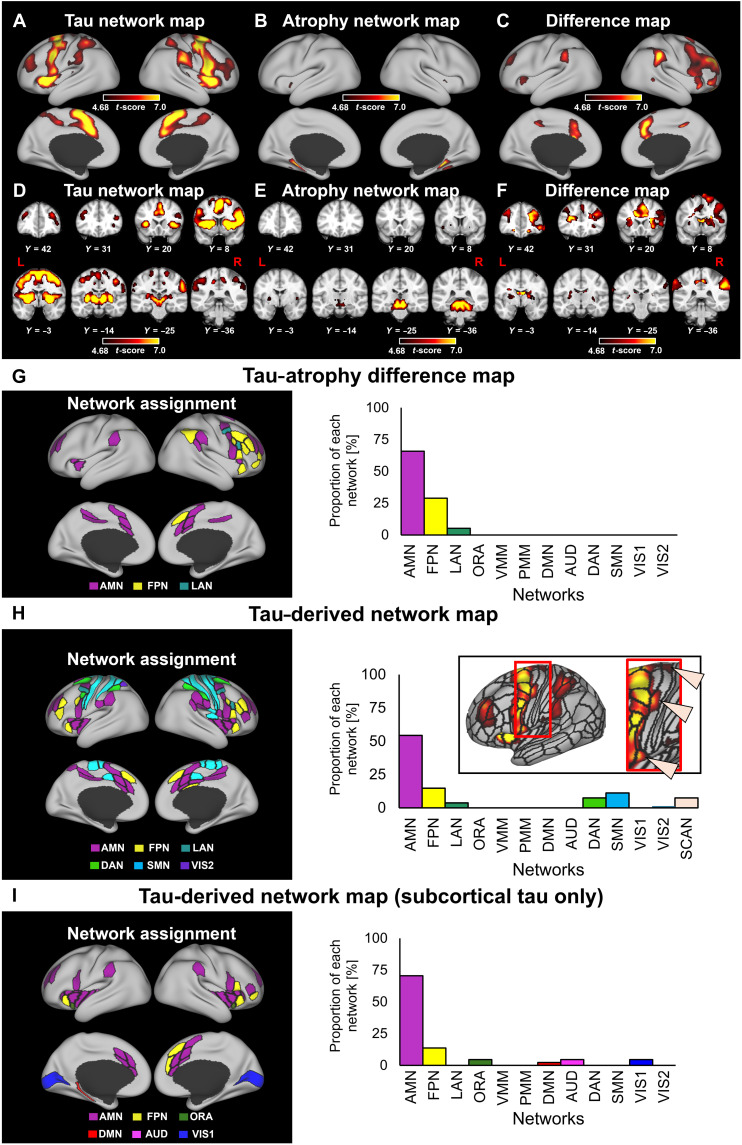
Tau and atrophy network maps for the patients with PSP. (**A** to **F**) Population network maps calculated using tau deposit and atrophy VOIs in individual patients as seed regions were presented on the surface [(A and B) for tau and atrophy, respectively] and volume space [(D and E) for tau and atrophy, respectively]. The difference map was obtained by comparing normative FC maps seeded from tau deposition sites and atrophy sites (C and F). (**G** to **I**) Overlap analysis with canonical functional networks. On the basis of the cortical parcellation in (*43*), 1 of 12 canonical networks was assigned to each region in the difference map (G), the tau network map before comparison with atrophy network map (H), and the tau network map calculated from subcortical tau deposits (I). The proportion of each network area to the total area was calculated for the difference map (G), the tau network map before comparison with atrophy network map (H), and the tau network map calculated from subcortical tau deposits (I). In the top right of (H), the tau network map focusing on the motor cortex is shown, with three triangles indicating SCAN regions. AMN, action-mode network; FPN, frontoparietal network; LAN, language network; ORA, orbitoaffective network; VMM, ventral multimodal network; PMM, posterior multimodal network; DMN, default mode network; AUD, auditory network; DAN, dorsal attention network; SMN, somatomotor network; VIS1, visual network 1; VIS2, visual network 2; SCAN, somatocognitive action network.

Next, we sought to determine whether common networks functionally connected from tau deposit and/or atrophy locations could be observed across the patients. We first examined the brain regions that were functionally connected from the tau deposit locations in each patient using a tau network mapping approach (see fig. S3). The locations of tau deposit in each patient were identified on the basis of the *t*-map (*P* < 0.001, two-sided, uncorrected). These locations then served as seed regions for calculating the whole-brain normative FC maps using a publicly available resting-state functional MRI (rs-fMRI) dataset of healthy individuals [Brain Genomics Superstruct Project (GSP) (*41*, *42*)] (*n* = 100) (*27*). Although tau deposits were largely confined to subcortical regions and to limited portions of the motor-related cortex (Fig. 2, A and C), the analysis revealed a common network derived from tau deposits that extended to widespread cortical areas, including the lateral prefrontal cortex (LPFC), dorsal anterior cingulate cortex (dACC), posterior parietal cortex (PPC), and anterior insula (AI) [*P* < 0.05, family-wise error (FWE)–corrected; Fig. 3, A and D]. In contrast, atrophy network mapping based on the thresholded (*P* < 0.001, uncorrected) atrophy maps from the same patients revealed a common extended network largely restricted to subcortical regions, including the midbrain, pons, and a part of the cerebellum (Fig. 3, B and E). These results suggest that the extended, common cortical network for the patients with PSP can be derived specifically from tau deposits, rather than atrophy.

To validate these results, we conducted several control analyses as follows. First, we confirmed that the observed common network was largely preserved even when more liberal or stringent statistical thresholds were applied to define the seed regions from the tau accumulation and atrophy maps (fig. S4), suggesting that the results did not depend on a specific threshold value for seed definition. Second, reproducibility of the common cortical network was assessed by randomly half-splitting the original dataset into two subgroups. The resultant normative FC maps derived from tau deposits or atrophy were significantly correlated between the two subgroups (fig. S5, A to C and E to G; *r* = 0.87 and 0.73, *P* < 0.001 for tau network mapping and atrophy network mapping, respectively). We further randomly divided 100 times the dataset into two subgroups. The obtained mean correlation coefficients were statistically significant for both tau and atrophy network maps (*r* = 0.92 and 0.88, *P* < 0.001 for tau and atrophy, respectively), well exceeded the 95th percentile of the correlation coefficients calculated from shuffled pairs of FC values (fig. S5, D and H). The results indicate high and significant reproducibility of the obtained network maps. Third, to rule out the possibility of connectome-specific effects, we calculated the tau deposit-derived network using an alternative publicly available rs-fMRI database, the Human Connectome Project (HCP) (*43*). The cortical nodes identified in this analysis were similar to the original ones derived using the GSP database (fig. S6), and the resultant networks were significantly correlated with each other (Spearman’s ranked rho = 0.30, *P* < 0.001), supporting the robustness of our findings across different rs-fMRI datasets. Fourth, to ensure that the resultant networks were specific to the tau deposit or atrophy locations and not merely reflected “hub” regions in the brain, we contrasted the tau and atrophy network maps against corresponding control maps derived from spatially randomized seeds (*44*) with the same number of voxels as those of the original tau depositions across the patients. The results were consistent with the original tau and atrophy network maps (fig. S7), denying the possibility of reflecting hub regions on the network maps. These results confirmed that the observed common networks were specifically and reliably derived from tau deposit and atrophy locations in patients with PSP. Last, the patients’ ages were significantly higher than those of the HCs. Because tau deposition generally increases with age, the size of seed regions in the older patients might have been overestimated. Note that, however, a control analysis using different statistical thresholds for defining tau deposition seeds yielded largely similar network distributions (fig. S4). To further address the potential confounding effect of age, we performed an additional analysis using an age-matched PSP subgroup created by excluding the oldest seven patients with PSP. Tau network mapping for this age-matched subgroup yielded a spatial distribution highly similar to that obtained from the original cohort, with a significant spatial correlation between the two network maps (fig. S8). Together, these results indicate that the identified PSP-tau network is robust against the age difference between the groups and is not attributable to the inclusion of the older patients.

Because tau deposits are thought to cause neuronal loss and subsequent atrophy (*6*), another possible concern is that the observed tau deposit-derived network might partially reflect the remote effects of atrophy. To isolate the tau-specific extended common network, we further contrasted the tau- and atrophy-derived network maps from the same patients. This analysis revealed predominantly cortical nodes with significantly stronger connectivity from the tau deposit locations than from the atrophy locations, including the anterior dorsolateral PFC (adlPFC), dorsomedial frontal cortex (dmFC), dACC, premotor cortex (PMC), inferior frontal cortex (IFC), AI, and PPC (*P* < 0.05, FWE-corrected; Fig. 3, C and F). No regions showed the opposite pattern (i.e., significantly stronger connectivity from the atrophy locations than from the tau deposit locations), and most of the above cortical nodes did not exhibit significant tau deposition. We refer to this cortical network derived specifically from tau deposits as the PSP-tau network.

To assess whether the derived PSP-tau network corresponds to specific canonical functional networks in healthy individuals, we mapped the regions in the PSP-tau network onto 12 functional networks defined by Ji *et al.* (*45*) (Fig. 3G). The distribution of regions across the networks was significantly uneven (*P* < 1.0 × 10^−16^, chi-square test), with most regions were located in the action-mode network (AMN; 66%) (*46*), followed by the frontoparietal network (FPN; 29%), and language network (LAN; 5.3%) networks. No nodes from any of the other nine networks (orbitoaffective, ventral multimodal, posterior multimodal, default mode, auditory, dorsal attention, somatomotor, primary visual, and secondary visual networks) were included in the PSP-tau network (Fig. 3G). These results suggest that the cortical remote dysfunction caused by tau deposition in patients with PSP is predominantly linked to multiple but specific canonical networks, especially the AMN and FPN jointly supporting adaptive and goal-directed behavior (*47*). Similar network assignment was observed for the tau network map before contrasting with the atrophy network map (Fig. 3H; *P* < 1.0 × 10^−29^, chi-square test; the AMN and FPN were the primary and secondary networks, respectively). The somatocognitive action network (SCAN), which is strongly interconnected with the AMN (*48*) but not defined in (*45*), was also included in the tau network map but only before contrasting with atrophy network map (Fig. 3H, top-right inset). Critically, we also confirmed that a similar network assignment was obtained even when seed regions were restricted to subcortical tau deposits across all patients (Fig. 3I; *P* < 1.0 × 10^−24^, chi-square test; the AMN and FPN were the primary and secondary networks, respectively), suggesting that the PSP-tau network was derived not only from the cortical tau deposits.

### Normative FC strength from tau deposition sites to the PSP-tau network explained across-patient variability of cognitive decline

**Fig. 4.**
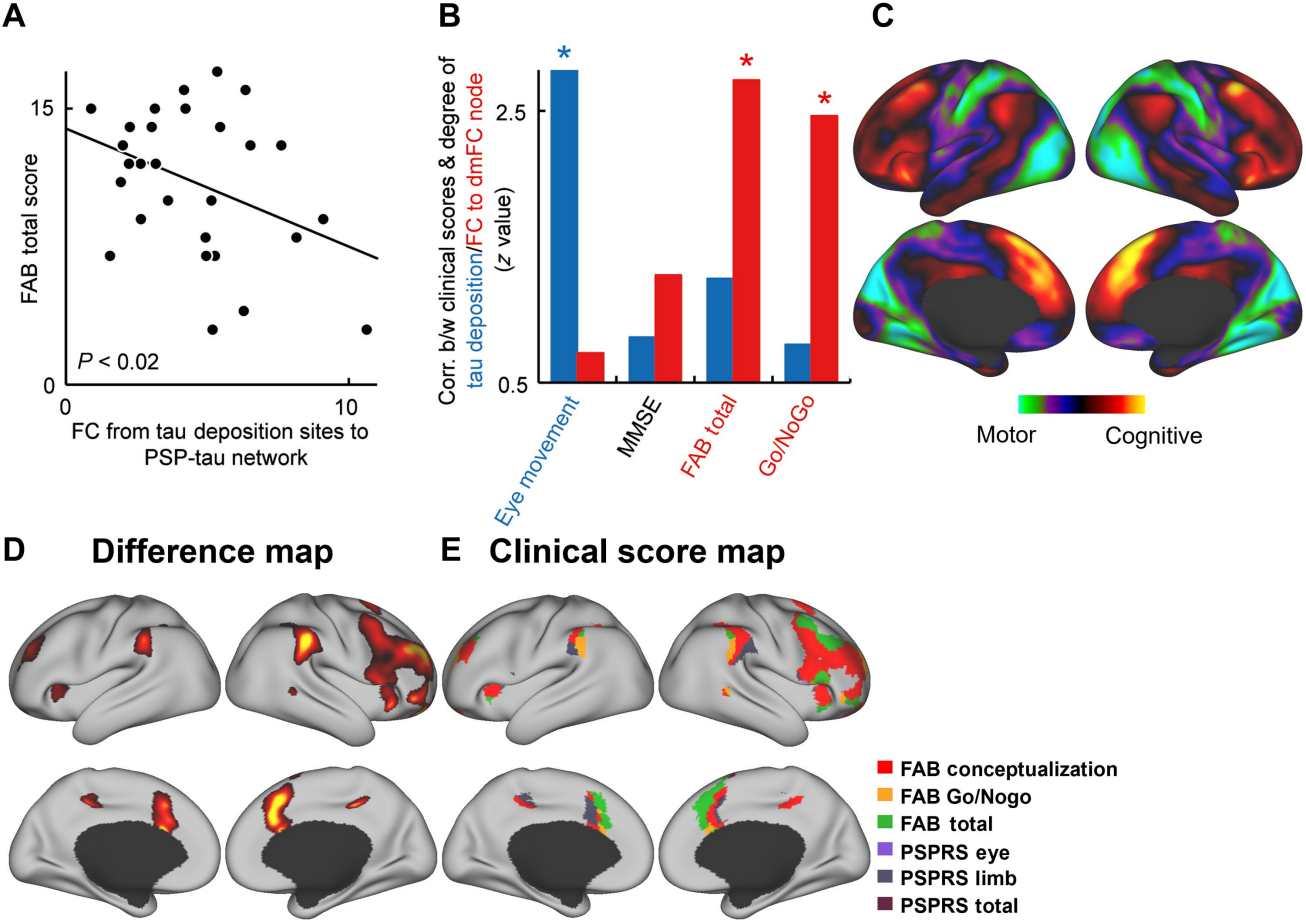
Normative FC strength from tau deposition sites to the PSP-tau network explained across-patient variability in cognitive decline. (**A**) Scatterplot showing that the normative FC value from tau deposition sites to the PSP-tau network exhibited significant negative correlation with the FAB total score across the patients (*P* < 0.02). (**B**) Correlations between motor/cognitive clinical scores and the degree of tau deposit in GP (blue) or normative FC value from tau deposition sites to dmFC node of the PSP-tau network (red). *P < 0.05, FDR-corrected for multiple comparisons. Note that the signs of *z* values for the MMSE, FAB total, and Go/NoGo task scores were inverted for display purposes. (**C**) Brain maps showing the regions whose normative FC values from tau deposition sites primarily explained motor (PSPRS limb) or cognitive (total FAB score) dysfunction. (**D** and **E**) The clinical scores with the strongest correlations were assigned to the brain regions in the PSP-tau network (D; same as Fig. 3C) using a winner-take-all method (E).

To assess how the derived PSP-tau network is involved in specific clinical symptoms at the single-patient level, we lastly examined the relationship between clinical scores and the normative FC strength from tau deposition sites to the PSP-tau network in the same patients, a proxy of estimated remote effects of tau deposition exerted on the network. In particular, we focused on whether such remote effects explain cognitive symptoms. The normative FC values from tau deposition sites to the PSP-tau network significantly explained the variance in the Frontal Assessment Battery (FAB) score across the patients, a general clinical test for measuring cognitive functions of the frontal cortex (*4*) (Fig. 4A) (*P* < 0.02). The result was consistent when multiple regression analysis was conducted with the degree of tau deposition in the same network and the local degree of tau deposition in the GP, where both the maximum overlap and the highest *t* value of tau deposition were observed across the patients as additional explanatory variables (*P* < 0.02). Conversely, these additional variables themselves did not explain the FAB score (*P* > 0.1). More closely, FC values from tau deposition sites to the dmFC node of the PSP-tau network in individual patients, a primary node in the frontal cortex without significant tau deposition (Fig. 3C), significantly explained the FAB score [*P* < 0.03, false discovery rate (FDR)–corrected for multiple comparisons] and its subscore Go/NoGo task performance (*P* < 0.05) (Fig. 4B, red), which requires response inhibition and whose impairment is well recognized as a primary symptom in patients with PSP (*49*, *50*). In addition, the same FC value also significantly explained the FAB subscore of conceptualization (*P* < 0.03, FDR-corrected), which has been causally linked with the dlPFC dysfunction (*51*). Meanwhile, the same normative FC values did not explain more general cognitive abilities indexed by the Mini-Mental State Examination (MMSE) (*P* > 0.4) or deficit of eye movement, one of the most typically impaired motor functions in patients with PSP (*1*, *52*) (*P* > 0.2) (Fig. 4B, red). The degree of tau deposition in the GP, in contrast, significantly explained the deficit in eye movement (*P* < 0.03, FDR-corrected) but did not explain the MMSE or any of the above cognitive scores (*P* > 0.2) (Fig. 4B, blue). A similar pattern was also observed for tau deposition in the midbrain, the secondary peak of tau deposition across the whole brain. To evaluate the relationship between local tau burden and frontal lobe–related cognitive functions more systematically, we additionally conducted a voxel-wise regression analysis across the whole brain, in which we correlated the FAB score with the extent of local tau deposition, specifically focusing on regions showing significant tau accumulation in each patient. Critically, however, no brain regions exhibited a significant association between local tau burden and the FAB score, whereas regions in which the normative FC value from tau deposition sites was significantly associated with the FAB score substantially overlapped with the PSP-tau network (fig. S9). These findings further support the notion that frontal lobe–related cognitive dysfunction in PSP is more closely related to the network-level effects of tau pathology than to local tau burden per se.

To map the regions where normative FC values from the tau deposition sites predominantly explains the cognitive impairments compared to the motor symptoms among the whole brain, we next constructed a map contrasting the correlations between normative FC and cognitive scores with those between normative FC and motor scores (Fig. 4C and figs. S10 and S11). After computing correlation maps for each of the FAB/PSPRS scores and their subscores (figs. S10 and S11), the scores showing the strongest association with normative FC values (total FAB score and the PSPRS subscore of limb movement deficit) were adopted to represent cognitive and motor symptoms. The brain areas where normative FC values from the tau deposition sites predominantly explained the cognitive deficits were the adlPFC, dACC, PMC, IFC, AI, and PPC, all of which were consistent with the PSP-tau network (Fig. 4, C and D) despite that these analyses were operationally independent of each other. The above contrast map significantly correlated with the PSP-tau network (*r* = 0.78, *P* < 0.001). Only the correlation maps for cognitive scores (i.e., FAB total, conceptualization, and Go/NoGo task) substantially overlapped with the PSP-tau network. To visualize the most relevant clinical score for each region in the PSP-tau network, we lastly constructed a clinical score map by assigning the score that exhibited the highest correlation with the normative FC values from tau deposition sites. This analysis revealed that, across the PSP-tau network, normative FC from tau deposition sites was more robustly associated with cognitive impairments than with motor deficits in individual patients (Fig. 4E).

Together, these results suggest that, at the single-patient level, whereas the motor symptoms stem directly from the local effects of subcortical tau burden, the cognitive symptoms can be best explained by remote effects of tau deposits exerted across the PSP-tau network.

## DISCUSSION

Here, we conducted tau network mapping and atrophy network mapping for patients with PSP based on multimodal imaging consisting of structural MRI and tau-PET with our recently developed high-contrast tracer florzolotau. Although tau deposits and atrophy were primarily detected in subcortical regions, the derived PSP-tau network was predominantly distributed across the cortical AMN and FPN. This network was derived specifically from tau deposition but not from atrophy. Critically, whereas the across-patient variance in motor symptoms was significantly explained directly by local effects of subcortical tau deposits, the cognitive symptoms were significantly explained only by remote effects of tau deposits exerted on the cortical PSP-tau network. These results suggest that the functional impacts of tau deposits in heterogeneous regions across patients with PSP remotely converge onto the cortical PSP-tau network, leading to their characteristic cognitive symptoms.

Network mapping with normative rs-fMRI data from a large population of healthy individuals has been extensively conducted for brain lesions especially from patients with stroke (*53*), and recently applied to atrophy (*35*) and tau deposits (*36*) in patients with AD. The present study effectively applied this approach to a non-AD tauopathy, with critical demonstration of both the distinction from random seed or atrophy-derived network maps and the dissociation between the influence of local tau deposits and their remote network effects on specific symptoms (*44*). In particular, the severity of cognitive, but not motor, symptoms in individual patients could be specifically explained by the normative FC strength from the tau deposition sites to the derived PSP-tau network, a proxy for the degree to which tau deposits exert functional impact on these remote regions in each patient. In contrast, a local amount of tau in the subcortical regions, where both the *t* value and the overlap across patients were prominent, could only predict motor deficits. These results demonstrate the significance of tau network mapping for elucidating brain-symptom relationships in patients with non-AD tauopathy. The current results indicating remote effects of tau burden on particular symptoms is consistent with the notion that cortical dysfunctions in patients with PSP do not result directly from local tau deposits but rather stem from network disruption that are remotely triggered by subcortical tau accumulation (*24*). Although the current use of tau network mapping itself did not specifically focus on cognitive deficits, the resultant strength of the normative FC from tau deposition sites to the derived PSP-tau network specifically explained frontal lobe–related cognitive aspects of the symptoms, rather than motor dysfunctions or more general cognitive disabilities. This is likely because the derived PSP-tau network included frontal cortical nodes and largely overlapped with the AMN and FPN as discussed in a later section.

Tau deposition sites were not entirely heterogeneous across the patients with PSP but were partially overlapped, especially in the GP and midbrain. These regions have been shown to exhibit significant positive FC and direct/indirect anatomical connectivity with some nodes in the PSP-tau network (*22*, *54*). This might raise a concern that the derived PSP-tau network merely reflected the normative FC map seeded from a specific primary region of tau deposit. However, correlations with clinical scores showed substantially different patterns between the amount of tau deposited in each of these regions and the strength of the normative FC from the tau deposition sites to the PSP-tau network (Fig. 4). This suggests that the derived PSP-tau network was not merely a simple reflection of the normative FC pattern from a specific single brain region with highly overlapped tau depositions across the patients but instead presumably reflected the normative FCs from multiple nodes where tau deposits were distributed heterogeneously across the patients.

It is also important to note that tau deposition in the patients with PSP was not exclusively confined to subcortical regions (Fig. 2A and fig. S2). However, when considering the group-averaged distribution and spatial overlap of tau deposition sites across patients, tau accumulation was substantially more consistent and prominent in subcortical regions than in the cortex. Moreover, when the tau network mapping was conducted using seeds restricted exclusively to subcortical tau deposition sites, the resultant PSP-tau network remained largely unchanged and still exhibited the largest overlap with the AMN, followed by the FPN. This indicates that subcortical tau pathology alone is basically sufficient to account for the convergent cortical network effects identified in the present study. Together, these findings suggest that subcortical tau pathology plays a dominant role in driving the network-level vulnerability associated with cognitive symptoms in PSP.

Whereas tau network mapping revealed a prominent convergent cortical network, an atrophy-derived similar approach for the same patients did not, even across a range of statistical thresholds for defining seed regions. This dissociation likely reflects both methodological and biological factors. Methodologically, tau-PET and structural MRI differ in their sensitivity, whereas biologically, tau deposition precedes and drives atrophy in PSP (*6*). Consequently, tau deposition may be a more sensitive marker of the underlying pathology than atrophy, particularly in patients at earlier stages of the disease. Therefore, although the spatial distributions of tau deposition and atrophy partially overlap, tau deposition may be more sensitive than atrophy for detecting the commonly connected network across the patients. Note, however, that atrophy network mapping with a liberal threshold for defining seed regions significantly detected the AI region, one of the PSP-tau network nodes, as a convergent cortical area commonly connected from atrophy sites across the patients (fig. S4G), suggesting the effectiveness of atrophy network mapping and a commonality between the resultant network maps derived from tau deposition and atrophy. Overall, the results suggest a potential advantage of using tau deposits detected by florzolotau PET instead of atrophy captured by structural MRI to map a common core pathological network across patients with PSP and to clarify the brain-symptom relationships. Tau deposition itself should be toxic to neurons directly and indirectly and thus would lead to neuronal dysfunctions even without atrophy. The current results suggest the functional impacts of tau deposition itself via remote common network in the PSP. Note that atrophy network mapping itself has been successfully conducted in previous studies including those for more large cohorts of patients with AD (*35*) or schizophrenia (*55*). Therefore, more elaborated analysis of structural MRI data including normative modeling (*56*) with a larger cohort might improve the resultant atrophy network map for the PSP in future research.

Previous studies have shown significant changes of functional and anatomical connectivity in patients with PSP compared to HCs. In particular, corticosubcortical changes in FC, including those between the midbrain/thalamus and cortical areas, have been reported (*22*, *57*). Cortical targets of such FC changes partially overlap with the now identified PSP-tau network. These FC changes in the patients should have also affected their FAB scores. However, relationships between such corticosubcortical FC changes and canonical functional networks or cognitive symptoms have not been examined, and the mechanistic difference between motor and cognitive symptoms have not been suggested. Furthermore, significant hypoperfusion and hypometabolism have been detected in the dACC, midcingulate cortex, and dlPFC of patients with PSP (*26*, *58*, *59*), which are located in spatial proximity to a subset of the PSP-tau network nodes and substantial tau burden has not been observed. Therefore, these physiological changes in patients with PSP might at least be partly explained by the tau deposition–derived extended network found in the present study.

Among the canonical networks, the PSP-tau network was most largely overlapped with the AMN (*46*), which is associated with planning, execution, monitoring, stopping of action, and attentional allocation. These functions of the network are well consistent with major symptoms of the PSP. Note that, however, the PSP-tau network was also overlapped with a part of the FPN, with which the AMN jointly supports adaptive and goal-directed behavior (*47*). This indicates that the PSP-tau network is not merely a reflection of a single canonical network but rather a composite of a few distinct networks, thereby exhibiting unique characteristics and association with clinical symptomatology. In particular, normative FC strength from tau deposition sites of each patient to the dmFC node in the PSP-tau network significantly explained the performance of a Go/NoGo task (Fig. 4), which requires the stopping of planned actions. The deficit in response inhibition is one of the primary symptoms observed in patients with PSP (*49*, *50*) and is likely reflected in the declined performance of a Go/NoGo task. Furthermore, not only the dmFC node but also a majority of nodes in the PSP-tau network are known to be recruited by tasks requiring response inhibition (*60*), and the across-patient correlation map between the Go/NoGo task performance and the normative FC strength from tau deposition sites highly overlapped with the whole PSP-tau network. These results suggest that the PSP-tau network is composed of a unique set of AMN/FPN nodes, characterizing the cognitive symptoms of PSP, especially impaired response inhibition.

The present study did not examine whether subcortical tau depositions cause actual dysfunction of the cortical PSP-tau network or whether dysfunction of the PSP-tau network induces cognitive symptoms. Recently, manipulation of neural activity has become possible in nonhuman primates using chemogenetic techniques such as Designer Receptors Exclusively Activated by Designer Drugs (DREADDs) and Pharmacologically Selective Actuator Module/Pharmacologically Selective Effector Molecule (PSAM/PSEM) systems (*61*, *62*), and remote effects of neuronal silencing have been visualized at functionally connected regions with behavioral relevance (*63*, *64*). Therefore, causal validation and mechanistic understanding of the results obtained with tau network mapping via reverse translational interventions in nonhuman primates is an intriguing direction for future research.

Other major limitations of the present study are as follows. First, the present study did not include rs-fMRI data from the patients. Second, although within-cohort reproducibility of the PSP-tau network was confirmed in a control analysis, its validation across independent cohorts would be required for more rigorous testing of reproducibility. The third limitation is the cross-sectional design of the research. Longitudinal tau-PET study tracking changes in the PSP-tau network alongside symptom progression will provide a causal understanding of the relationships between the PSP-tau network and cognitive symptoms. Fourth, although PSP can present asymmetric pathology, we did not explicitly assess hemispheric lateralization of tau deposition or the resultant PSP-tau network. Last, local tau burden in the PFC can be observed when broader PSP subtypes are included in the cohort (*65*). In the present study, however, we included only the patients with PSP-RS, and no such tau burden in the PFC or its association with cognitive symptoms was observed (Fig. 2A and fig. S9B). Instead, cognitive symptoms were well explained by the normative FC from tau deposition sites to the PSP-tau network. In these patients, tau burden may propagate from the epicenter along the connectivity as the disease progresses (*21*), at which point the cognitive symptoms may become explainable by local tau accumulation. Therefore, the balance between the local and remote effects of tau/atrophy will vary depending on both the cohort characteristics and the stage of the disease.

The present study demonstrated that the tau network mapping was able to identify a common cortical network to which tau deposition sites across patients with PSP are functionally connected. Tau-PET images obtained from patients with PSP can thus be applied to identify the relationships linking the predominantly subcortical tau deposits with the remote dysfunction of the connected cortical networks and the resultant cognitive symptoms. The current approach provides a potential target for symptom-specific neuromodulatory treatment for patients with PSP and might inform the future risk of cognitive decline for patients in the early phase of the disease, based on still subthreshold tau burden at brain regions connected to the PSP-tau network. Furthermore, this approach would be also widely appliable to other tauopathies including chronic traumatic encephalopathy (*66*) and late-onset psychiatric disorders (*67*), for which tau deposits can be reliably detected with florzolotau, as well as for other proteinopathies such as α-synucleinopathies (*68*), or other PET ligands measuring synaptic density or inflammation, to understand the mediating networks between the PET-detectable local pathology and the resultant symptoms in a range of brain diseases.

## MATERIALS AND METHODS

### Participants

Forty-six patients with PSP-RS and 50 HCs aged older than 40 years, without histories of neurological disorders were recruited from our affiliated hospitals and the volunteer association of our institute, respectively. Patient details were described in our previous paper (*40*), but PSP participants with PSP-tau scores less than 0.3 and HCs with PSP-tau scores more than 0.1 were excluded from the later analyses. The PSP-tau score is a diagnostic index derived from whole-brain florzolotau PET data using machine learning, reflecting both the spatial distribution and severity of PSP-RS–related tau pathology (*40*). The final numbers of participants included were 37 and 48 for patients and HCs, respectively. Gender was matched between patients and HCs (*P* > 0.05, chi-square test; table S1). The participants underwent several neurological examinations, including the PSPRS, FAB, and MMSE. All patients and HCs were negative for ^11^C-PiB-PET based on visual inspection of the acquired images by more than two experts. This study was approved by the Radiation Drug Safety Committee and the National Institutes for Quantum Science and Technology Certified Review Board of Japan (protocol/approval numbers 16-037, 17-027, 16-036, 17-034, and 20-035). Written informed consent was obtained from all participants in accordance with the Declaration of Helsinki. The study was registered with the UMIN Clinical Trials Registry (numbers 000026385, 000029608, 000026490, 000030248, and 000043458).

### PET and MRI data acquisition and preprocessing

Florzolotau PET images were acquired to detect tau deposition using a Biograph mCT flow system (Siemens, Erlangen, Germany) with 2-mm isotropic voxels. T1-weighted (T1w) images were acquired using a 3T MRI scanner (MAGNETOM Verio, Siemens, Erlangen, Germany) for registering PET images to the standard MRI space and evaluating atrophy [repetition time (TR) = 2300 ms; echo time (TE) = 1.95 ms; inversion time (TI) = 900 ms; flip angle = 9°; field of view (FOV) = 250 mm; matrix size = 512 × 512 × 176; slice thickness = 1 mm]. Detailed preprocessing was described in our previous paper (*40*). Briefly, standardized uptake value ratio (SUVR) images were generated from averaged PET images for each participant with motion correction at the intervals of 90 to 110 min after intravenous injection of ^18^F-florzolotau [186 ± 8.4 (megabecquerel)]. The gray matter reference region was the area identified by the histogram of tau accumulation as the region of low tau accumulation (*69*). Individual SUVR images were spatially normalized to the standard Montreal Neurological Institute (MNI) space (East Asian brain T1w image from the International Consortium for Brain Mapping) using the Diffeomorphic Anatomical Registration Through Exponentiated Lie Algebra (DARTEL) algorithm from Statistical Parametric Mapping (SPM12, Wellcome Department of Cognitive Neurology), running on MATLAB (MathWorks).

### Tau network mapping

Tau network mapping was conducted using normative resting-state FC data from healthy individuals instead of the FC data from the patients themselves. This approach was designed to estimate the targets of remote effects exerted by tau pathology based on the intrinsic functional architecture before tau accumulation, rather than connectivity patterns potentially altered in the disease state. In this study, normative FC maps were computed using rs-fMRI data obtained from 100 healthy participants (*27*) from the GSP (*41*, *42*) with tau deposition sites in individual patients as seed regions. Figure S3 shows the analytical pipeline for tau network mapping. In the first step, tau deposition sites for each patient were identified by comparisons with tau-PET images of HCs. The *t*-score maps for individual patients were thresholded at *P* < 0.001 (two-sided, uncorrected) and were binarized to create the volumes of interest (VOIs) for calculating normative FC maps. Next, the mean timeseries from the tau deposition sites (i.e., VOIs) of a given patient for rs-fMRI data of each participant were compared with the timeseries from every voxel to calculate the correlation map. The maps for all 100 rs-fMRI datasets were then combined to calculate the *t*-score map for each patient. As a control condition of tau network mapping, we also calculated *t*-score maps using spatially randomized VOIs (*44*). Briefly, the seeds with the same number of voxels as those of the original tau depositions were randomly selected across the whole brain. The *t*-score maps were then calculated using these spatially randomized VOIs in the same manner as the original *t*-score maps for tau deposition. Last, random control-subtracted tau-derived FC map was obtained by contrasting between the normative FC maps derived from tau deposition and the randomized seeds. Normative FC map calculation, statistical tests, and surface mapping were performed using the FMRIB Software Library’s (FSL’s) FEAT tool (*70*), Analysis of Functional NeuroImages (AFNI) software (*71*), and Connectome Workbench (*72*), respectively.

### Atrophy network mapping

Data preprocessing for VBM was performed using SPM12 on MATLAB and PMOD 4.2/4.3 (PMOD Technologies LLC, Switzerland). Gray and white matter segmentation images were extracted from T1w images and combined to obtain the parenchymal image using SPM12. The DARTEL algorithm was then used to spatially normalize each brain parenchymal image (gray and white matter) by preserving amounts in the MNI space. The regions of atrophy for each patient were identified by comparisons with parenchymal images of HCs. The *t*-score maps for each patient were thresholded at *P* < 0.001 (two-sided, uncorrected) and were binarized to create the VOIs for calculating normative FC maps. Later analyses were the same as those for the tau network mapping described above.

### Assignments of canonical networks to regions in the derived PSP-tau network

On the basis of the atlas in Ji *et al.* (*45*), 1 of 12 canonical networks including the orbitoaffective, ventral multimodal, posterior multimodal, default mode, auditory, frontoparietal, language, dorsal attention, cingulo-opercular [action-mode (*46*)], somatomotor, and primary and secondary visual networks were assigned to each brain area in the tau network map before comparison with atrophy network map and the PSP-tau network that was determined as significant (*P* < 0.05, FWE-corrected) regions in a statistical comparison (paired *t* test) between the normative FC maps derived from tau and atrophy network mapping among the patients. Furthermore, we also assigned the canonical networks to each brain area in the tau network map, calculated based solely on subcortical tau deposits across all patients. The proportion of the number of areas to which each network assigned was then calculated and statistically evaluated using a chi-square test. To identify the SCAN within the somatomotor network, we used three coordinates of intereffectors in the superior, middle, and inferior parts of the primary motor areas reported by Gordon *et al.* (*48*).

### Validation analyses of tau/atrophy network mapping

To examine the consistency/stability of the tau/atrophy network mapping results among different criteria for the seed definition, both more liberal (*P* < 0.005, uncorrected) and more stringent (*P* < 0.05, FWE-corrected) thresholds compared with the original one (*P* < 0.001, uncorrected) were also tested to identify the locations of tau deposition or atrophy in the patients for subsequent network mapping (fig. S4). To confirm the reproducibility of tau/atrophy network mapping in the current cohort of patients, tau/atrophy network maps calculated for individual patients (*N* = 37) were randomly divided 100 times into two groups, and the tau/atrophy network mapping was performed for these two groups separately. Then, the resultant normative FC values (from the tau deposition sites) to the Johns Hopkins University (JHU) whole-brain VOIs were compared between the two groups. We assessed statistical significance by testing whether the mean correlation coefficients between the two groups exceeded the 95th percentile of the correlation coefficients obtained for pairs of randomly shuffled regions of interest (ROIs) (fig. S5). To confirm the consistency of tau network mapping results among different rs-fMRI databases, we also performed tau network mapping based on the HCP (*43*) data. For each patient, the normative FC map seeded from the tau deposition sites was calculated with the HCP young adult dataset (*n* = 98) in the same manner as the analysis using the GSP dataset described above. The voxels with top 10% normative FC values in the cortex were then extracted from the resultant tau network maps based on GSP/HCP data for comparison between the two rs-fMRI datasets. Normative FC values in the JHU whole-brain VOIs were also calculated to quantify the threshold-free correlation between the maps obtained with GSP/HCP data (fig. S6).

### Across-patient correlation between clinical scores and normative FC values from tau deposition sites to the PSP-tau network

To complement the single regression analysis showing that the FAB total score could be significantly explained by the normative FC strength from tau deposition sites to the PSP-tau network (Fig. 4A), multiple regression analysis was further conducted using an R package, in which additional control explanatory variables were the degree of tau deposition in the GP, where both the maximum overlap and the highest *t* value of tau deposition across the whole brain were observed, and in the PSP-tau network itself. To more closely examine the relationships between the severity of cognitive/motor symptoms and the remote/local effect of tau burden across the patients (Fig. 4B), we then extracted the normative FC strength from tau deposition sites to the dmFC node of the PSP-tau network, a representative frontal node in the network and the amount of tau deposition in the GP. Pearson’s correlation coefficients were then calculated between these values and clinical scores (PSPRS and FAB) including their subscores. The MMSE served as a control score reflecting general cognitive ability. Statistical significance was assessed using FDR correction for multiple comparisons.

To visualize brain regions where local tau deposition or normative FC from tau deposition sites was associated with clinical symptoms at the individual level, we additionally performed voxel-wise regression analyses using the amount of tau deposition or normative FC from tau deposition sites as a regressor to predict clinical scores (PSPRS and FAB). This yielded whole-brain maps identifying regions where interindividual variability in tau deposition or normative FC strength was associated with variability in clinical symptoms (figs. S9 to S11). To visualize the regions where normative FC from tau deposition sites predominantly explained the cognitive impairments against motor-related dysfunctions at the whole-brain level (Fig. 4C), we contrasted the whole-brain correlation map between the FC and the cognitive symptoms against that between the normative FC and motor deficits. On the basis of the maximum values of correlation coefficient between the normative FC strength and clinical scores, the total score of the FAB and the limb movement deficit score of the PSPRS were adopted as indices representing cognitive and motor impairments, respectively. Last, to visualize the clinical score that most strongly correlated with the normative FC value from tau deposition sites to each brain region within the PSP-tau network (Fig. 4E), the whole-brain correlation maps between the normative FC value from tau deposition sites and individual clinical scores were calculated, and the clinical scores with the strongest correlation were assigned to each brain region of the PSP-tau network. The above whole-brain analyses were performed using the SPM12, Connectome Workbench, and FSL software.
