# Supplementary material for: Remote network for cognitive symptoms derived from tau accumulation in progressive supranuclear palsy
Source: Sci Adv. 2026 Jul 10;12(28):eaed0348. doi: 10.1126/sciadv.aed0348 (PMC13353357; doi:10.1126/sciadv.aed0348)
Supplement: Supplementary file 1 — Figs. S1 to S11 Table S1 [file sciadv.aed0348_sm.pdf]

Science Advances
AAAS

# Supplementary Materials for

## Remote network for cognitive symptoms derived from tau accumulation in progressive supranuclear palsy

Yuki Hori *et al.*

Corresponding author: Hironobu Endo, endo.hironobu@qst.go.jp;
Toshiyuki Hirabayashi, hirabayashi.toshiyuki@qst.go.jp

*Sci. Adv.* **12**, eaed0348 (2026)
DOI: 10.1126/sciadv.aed0348

**This PDF file includes:**

Figs. S1 to S11
Table S1

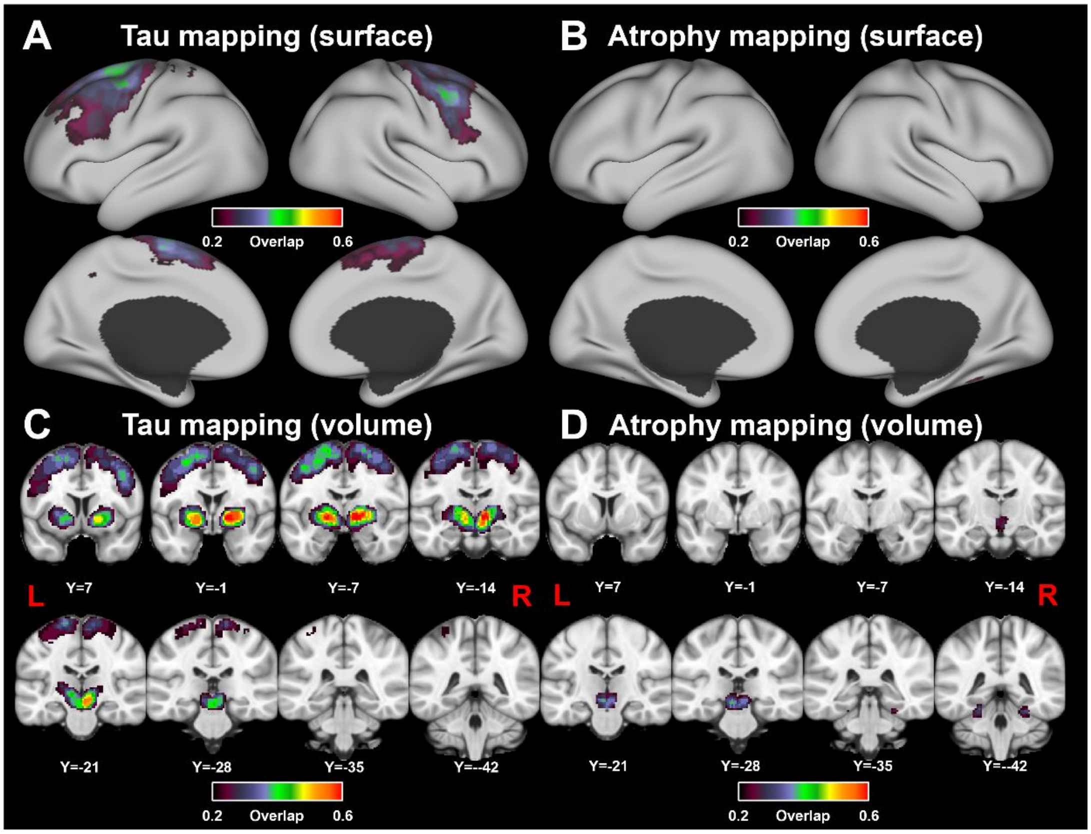


**Fig. S1. Spatial overlap of tau deposit and brain atrophy locations among the patients with PSP.** Spatial overlap maps for tau deposition [(**A**) and (**C**)] and atrophy [(**B**) and (**D**)] are presented on the surface [(A) and (B)] and in volume space [(C) and (D)]. Significant tau accumulation was observed in the GP (thresholded at $p < 0.001$, overlapped maximally for 62% of patients), midbrain (57%), and parts of motor-related cortex (25%), while brain atrophy was significantly observed in the midbrain (35%) and cerebellum (32%).

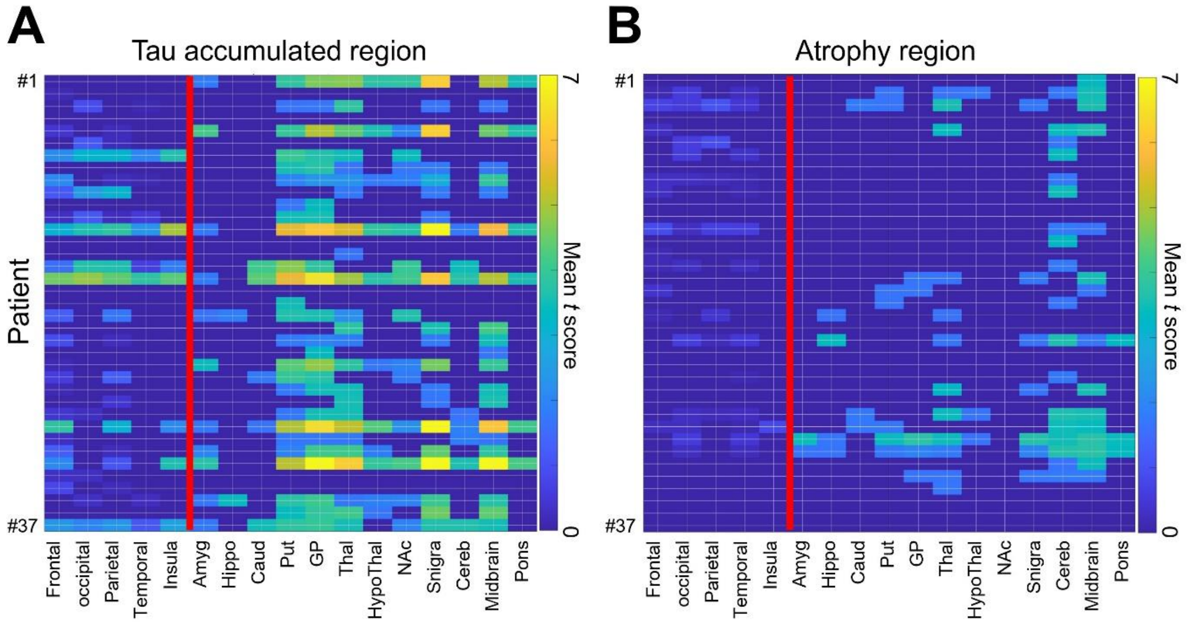


**Fig. S2. Distribution of tau deposition and atrophy across brain regions in individual patients with PSP.** Heatmap matrices illustrating the regional distribution and relative magnitude of tau deposition (**A**) and brain atrophy (**B**) for each individual patient. Rows correspond to individual patients, and columns represent predefined brain regions selected from representative whole-brain volumes of interest (VOIs) based on the Johns Hopkins University (JHU) atlas. Red lines delineate boundaries between cortical and subcortical regions. Color intensity indicates the degree of tau-PET uptake or atrophy within each region determined by unpaired *t*-test against healthy controls. The darkest blue, no significant ($p$ < 0.001, uncorrected) tau deposition or atrophy. *Amyg: Amygdala; Hippo: Hippocampus; Caud: Caudate; Put: Putamen; GP: Globus pallidus; Thal: Thalamus; HypoThal: Hypothalamus; NAc: Nucleus accumbens; Snigla: Substantia nigra; Cereb: Cerebellum.*

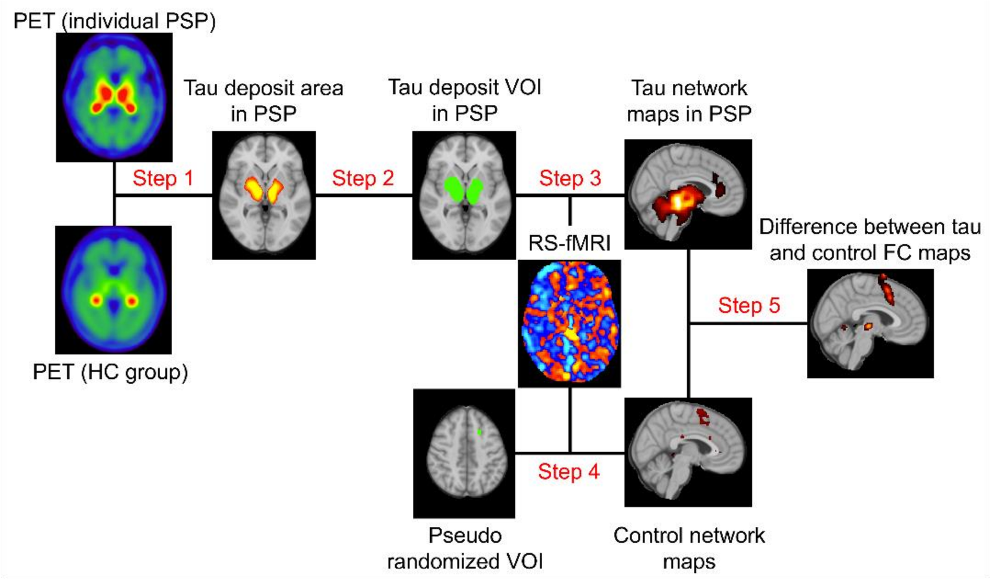


**Fig. S3. Analytic pipeline for tau-network mapping.** Tau deposition sites for each patient were identified by comparisons with tau PET images from healthy controls (Step 1). The *t*-score maps of tau deposition for individual patients were then thresholded at $p < 0.001$ (two-tailed, uncorrected), and binarized to create the VOIs (Step 2). Next, the mean timeseries in the VOI from each rs-fMRI data ($n$ = 100) were compared with timeseries for every voxel to calculate the correlation maps. The maps for all 100 rs-fMRI datasets were combined to calculate the *t*-score map for each patient (Step 3). As a control, *t*-score maps were calculated using spatially randomized voxels in the same manner as using the tau deposit VOIs (Step 4). Finally, tau-derived FC map was obtained by calculating a differential *t*-score map between tau deposition-derived and control FC maps across the patients (Step 5).

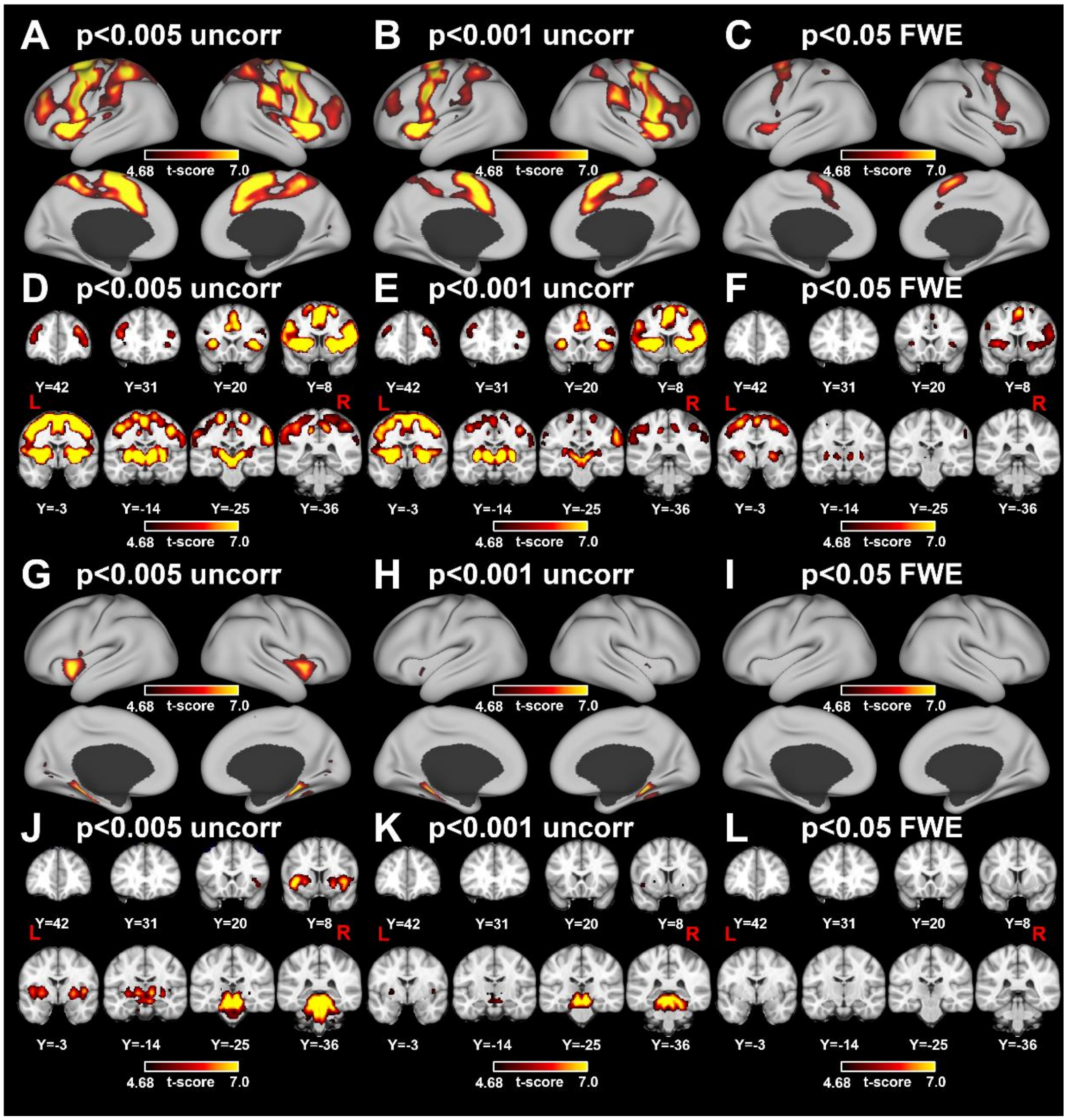


**Fig. S4. Tau/atrophy network maps with different thresholds for seed definition.** Tau- [(**A**) to (**F**)] and atrophy- [(**G**) to (**L**)] network maps were obtained using seed regions derived from tau deposits and atrophy with three different thresholds ($p < 0.005$ uncorrected, $p < 0.001$ uncorrected, and $p < 0.05$ FWE-corrected).

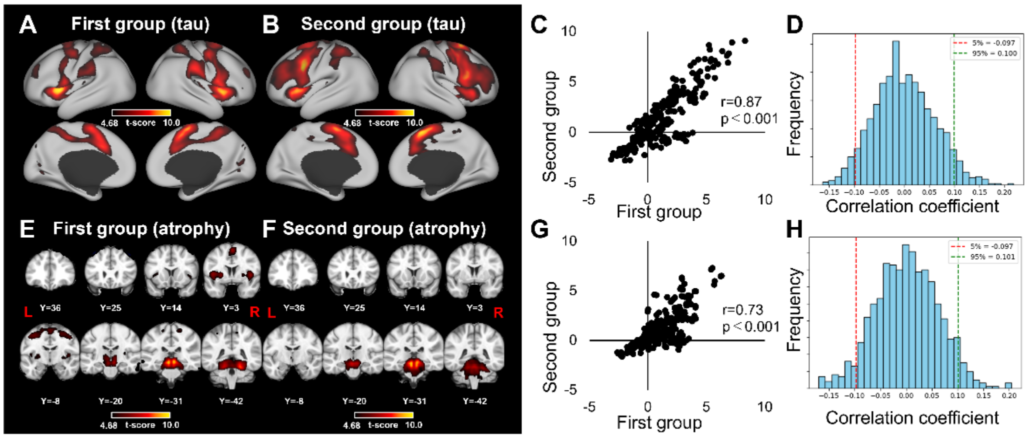


**Fig. S5. Reproducibility of tau/atrophy-network mapping.** (**A** to **D**) Comparisons of tau-network mapping results for split-half subgroups. Correlation of FC values for the same region was plotted in (C). The histogram of correlation coefficients obtained from randomly shuffled ROIs showed that the 5th percentile is -0.097 and the 95th percentile is 0.10 (D). (**E** to **H)** Same as [(A) to (D)], but for atrophy. Volume maps were adopted instead of surface maps for atrophy due to subcortical predominance. The VOI values in the tau/atrophy-network maps across the whole brain were highly consistent between the first and second groups of patients ($r = 0.87$ and 0.73, $p < 0.001$ for tau- and atrophy-network map, respectively). When all patients were randomly divided into two groups 100 times, the mean correlation coefficient ($r = 0.92$ and 0.88, $p < 0.001$ for tau- and atrophy-network map, respectively) exceeded the 95th percentile of the correlation coefficients obtained from randomly shuffled ROIs.

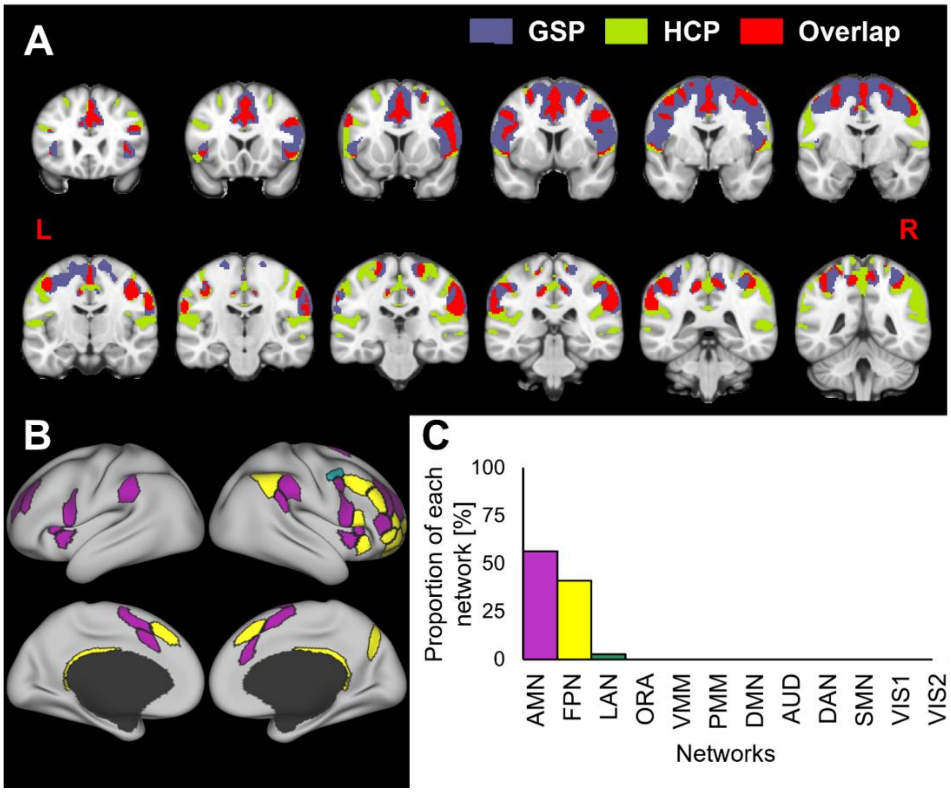


**Fig. S6. Consistency of tau-network mapping results across two different rs-fMRI databases.** (**A**) Tau-network maps were computed based on GSP and HCP databases. Voxels showing top 10% of the FC values in the cortex are shown in blue and green for GSP and HCP, respectively. Overlap between the tau-network maps based on the two databases were depicted in red, including the LPFC, dACC, PPC, and AI. (**B** and **C**) Similar to Fig. 3H, one of twelve canonical networks was assigned to each region in the tau-network maps computed using HCP database (B), and the proportion of areas assigned to each network was calculated (C).

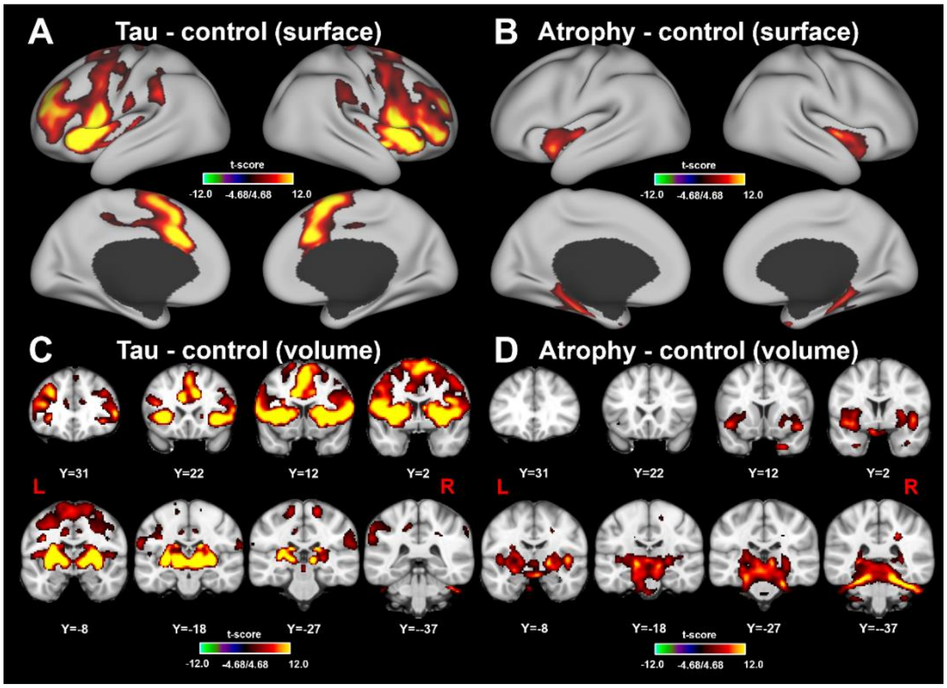


**Fig. S7. Comparison of tau/atrophy-network maps with control normative FC maps calculated with spatially randomized seed regions.** (**A** and **C**) Control-subtracted tau-network map presented in the surface- (A) and volume- (C) spaces, respectively. (**B** and **D**) Same as [(A) and (C)], but for atrophy-network mapping.

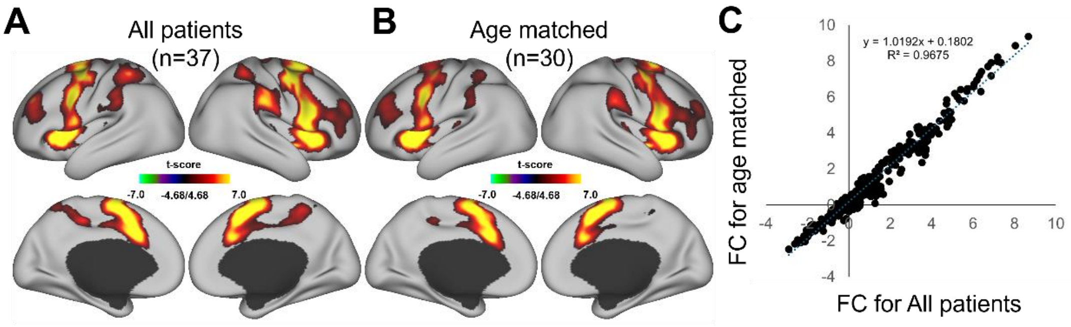


**Fig. S8. Robustness of tau network mapping against age differences.** (**A**) Tau-network map derived from all PSP patients included in the main analysis ($N = 37$), using tau-deposition seeds defined relative to the healthy control group. (**B**) Tau-network map derived from an age-matched PSP subgroup ($n = 30$) after excluding the seven oldest PSP patients to minimize age difference relative to the healthy controls. (**C**) Spatial correlation analysis between the tau-network maps obtained from the full PSP cohort (A) and the age-matched PSP subgroup (B). Each data point represents a VOI value shared between the two maps. The two network maps showed a very strong and statistically significant spatial correlation ($p < 1.0\times10^{-149}$), indicating that the identified tau-associated network architecture is robust to age differences between the groups.

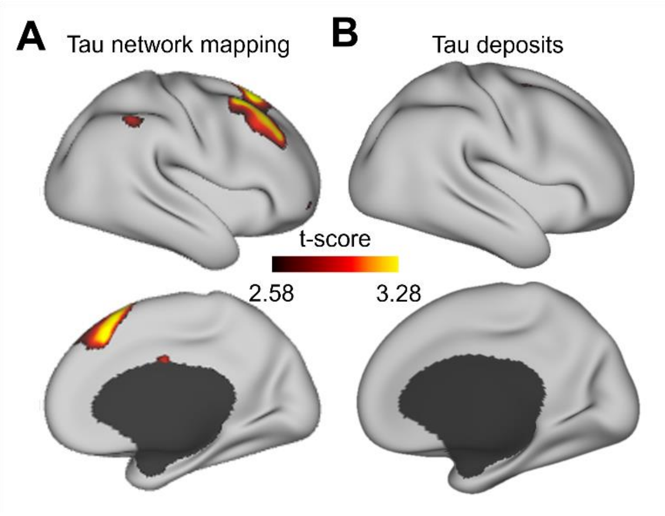


**Fig. S9. Brain regions in which the normative FC value from tau-deposition sites or the local extent of tau deposition explains cognitive dysfunction.** Surface maps showing brain regions in which either the normative FC value from tau-deposition sites (**A**) or the local extent of tau deposition (**B**) was associated with cognitive dysfunction at the individual level, as assessed by the total FAB score. Regions were identified based on voxel-wise correlations between the FAB total score and either the normative FC value from tau-deposition sites (A) or the local extent of tau deposition (B). Statistical maps were thresholded at $p < 0.01$ (uncorrected). Note that the signs of the correlation coefficients are inverted for display purposes.

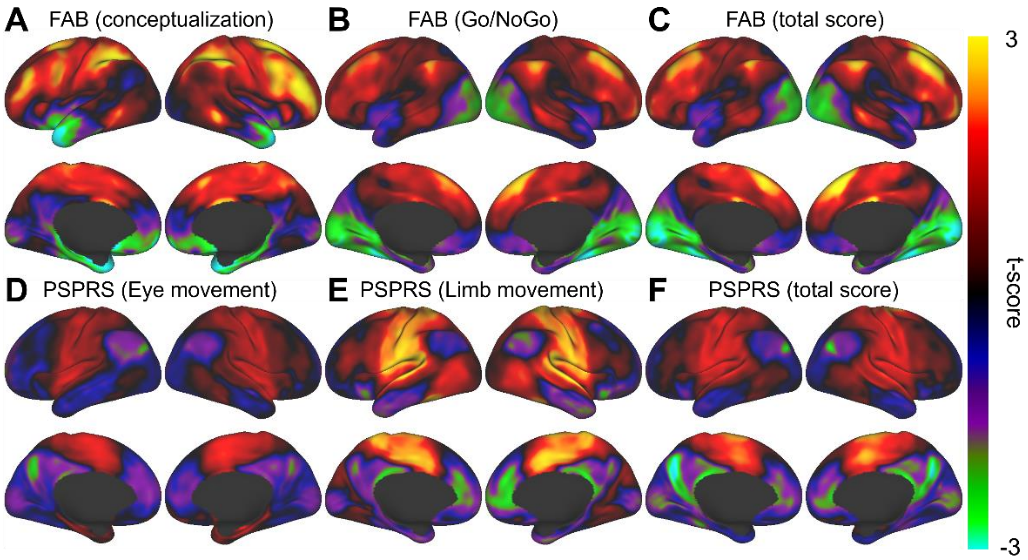


**Fig. S10. Correlation maps between the FC value from tau deposition sites and the degree of cognitive and motor symptoms.** Whole-brain voxelwise correlation maps show the associations between the tau-network map and (**A**) FAB conceptualization subscore, (**B**) FAB Go/No-Go subscore, (**C**) FAB total score, (**D**) PSPRS eye movement subscore, (**E**) PSPRS limb movement subscore, or (**F**) PSPRS total score. Note that the signs of the correlation coefficients in [(A) to (C)] are inverted for display purposes.

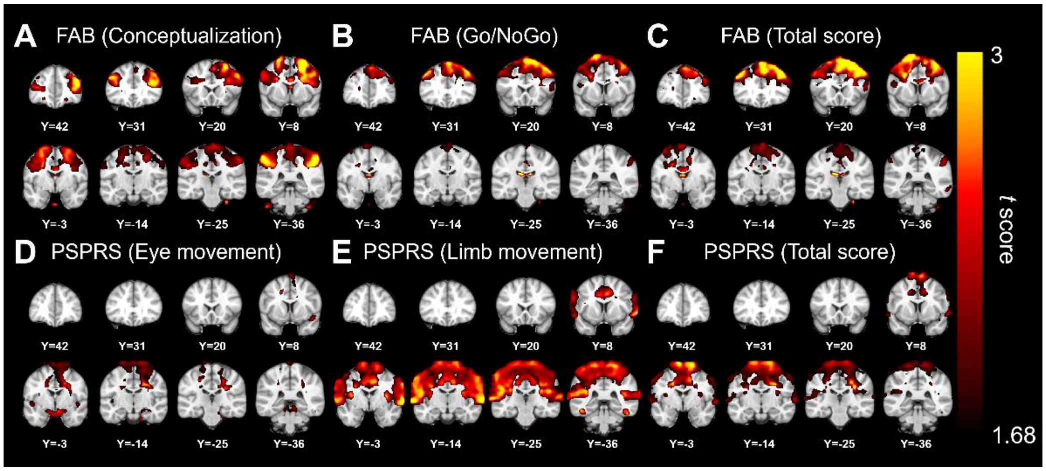


**Fig. S11. Correlation maps between the FC value from tau deposition sites and the degree of cognitive and motor symptoms in volume space.** Whole-brain (including subcortical regions) voxelwise correlation maps show the associations between the tau-network map and (**A**) FAB conceptualization subscore, (**B**) FAB Go/No-Go subscore, (**C**) FAB total score, (**D**) PSPRS eye movement subscore, (**E**) PSPRS limb movement subscore, or (**F**) PSPRS total score. Maps were threshold at $p < 0.05$ (uncorrected). Note that the signs of the correlation coefficients in [(A) to (C)] are inverted for display purposes.

**Table S1. Demographic and clinical characteristics of the study participants**. Values are presented as mean ± SD. *P*-values were calculated using Welch's *t*-test for age, chi-square test for gender, and two-sampled *t*-tests for the others. *; $p < 0.01$. HC, healthy control; PSP, progressive supranuclear palsy; PSPRS, PSP rating scale; FAB, Frontal Assessment Battery; MMSE, Mini-Mental State Examination.

| | HC (n=48) | PSP (n=37) |
|---|---|---|
| Age (year) | 64.1 ± 11.3 | 70.0 ± 7.3 * |
| Sex (female) | 21 | 15 |
| Disease duration (year) | - | 3.4 ± 2.4 |
| PSPRS | 1.6 ± 1.9 | 40.9 ± 17.4 * |
| FAB | 16.5 ± 1.2 | 11.2 ± 3.9 * |
| MMSE | 29.3 ± 0.9 | 23.4 ± 5.5 * |
